# An Instrument to Measure Maturity of Integrated Care: A First Validation Study

**DOI:** 10.5334/ijic.3063

**Published:** 2018-01-25

**Authors:** Liset Grooten, Liesbeth Borgermans, Hubertus JM Vrijhoef

**Affiliations:** 1 Department of Family Medicine and Chronic Care, Faculty of Medicine and Pharmacy, Vrije Universiteit Brussel, B-1090 Brussels, BE; 2 Department Patient and Care, Maastricht University Medical Center, Maastricht, NL; 3 Panaxea B.V., Amsterdam, NL

**Keywords:** integrated care, measurement, maturity, instrument, validity

## Abstract

**Introduction:**

Lessons captured from interviews with 12 European regions are represented in a new instrument, the B3-Maturity Model (B3-MM). B3-MM aims to assess maturity along 12 dimensions reflecting the various aspects that need to be managed in order to deliver integrated care. The objective of the study was to test the content validity of B3-MM as part of SCIROCCO (Scaling Integrated Care into Context), a European Union funded project.

**Methods:**

A literature review was conducted to compare B3-MM’s 12 dimensions and their measurement scales with existing measures and instruments that focus on assessing the development of integrated care. Subsequently, a three-round survey conducted through a Delphi study with international experts in the field of integrated care was performed to test the relevance of: 1) the dimensions, 2) the maturity indicators and 3) the assessment scale used in B3-MM.

**Results:**

The 11 articles included in the literature review confirmed all the dimensions described in the original version of B3-MM. The Delphi study rounds resulted in various phrasing amendments of indicators and assessment scale. Full agreement among the experts on the relevance of the 12 B3-MM dimensions, their indicators, and assessment scale was reached after the third Delphi round.

**Conclusion and discussion:**

The B3-MM dimensions, maturity indicators and assessment scale showed satisfactory content validity. While the B3-MM is a unique instrument based on existing knowledge and experiences of regions in integrated care, further testing is needed to explore other measurement properties of B3-MM.

## Introduction

Health systems around the world are under great pressure to drive forward transformation in order to meet the evolving needs of their populations. The traditional disease orientated approaches currently provided no longer suffice to meet the people’s needs [1]. In many countries care is too often fragmented and has clear deficiencies in quality, inducing low responsiveness of the health system and low satisfaction with health services [2]. To address these challenges, the transformation towards integrated care has the potential to repair deficiencies in order to obtain accessible, quality, effective and sustainable health care.

Integrated care initiatives are being developed around the world [3,4,5]. Countries in Europe have endeavoured to improve the performance of their health systems. Many have implemented different types of integrated care programmes representing a diversity in their nature and scope of approaches [6]. This is not surprising since the transition to integrated care is a complex undertaking, while sufficient support of a systematic understanding of integration in health systems is scarce [7,8,9]. The complexity of integration is reflected in the definition of integrated care provided by Kodner [10, p. 12]: “[a] multi-level, multi-modal, demand driven and patient-centred strategy designed to address complex and costly health needs by achieving better coordination of services across the entire care continuum. Not an end in itself, integrated care is a means of optimizing system performance and attaining quality patient outcomes.” As a response to the call for the establishment of a common language and framework of integrated care to better understand integrated care and guide empirical research [10,11], several studies have attempted to clarify the concepts underpinning integrated care [8,12].

In particular, the conceptual framework of Valentijn et al. can be used to aid an understanding of the concept of integrated care [8] and shows the complexity of what transformation to integrated care delivery entails. The framework identifies key elements for achieving integrated service delivery which are organised into six dimensions of integration. The features have complementary roles on the micro (clinical integration), meso (professional and organisational integration), and macro (system integration) levels to deliver comprehensive services that address the needs of people and populations. Functional and normative integration establish connectivity across the micro, meso and macro level. Transitioning to integrated care involves various related activities taking place at different levels of the health system and including diverse actors and organisations with various perspectives on integrated care.

To achieve a successful transformation to more integrated care systems, insights are needed into what factors contribute to the progress and success of integrated care interventions. However, there is a lack of substantiation of the working mechanisms in integrated care, partly stemming from poor or non-existent evaluation and measurement of integrated care interventions [13].

Capturing the complexity of measuring integrated care is a challenging task. A first difficulty is that measurement is complicated by the conceptual haziness adhering to integrated care which thus limits the theoretical foundations of existing instruments [14]. A second difficulty is the demonstration of association between the changes in services as part of the integration efforts’ and their outcomes [15,16,17]. The linkage between changes in services and service outcomes is problematic because most patient or service user outcomes do not emerge from linear cause and effect chains [18]. Current measures of quality in health care, such as the structure-process-outcome model, do not clarify the underlying mechanisms governing the components of integrated care [19,20,21,22]. A sound analytical method for evaluating the outcomes of integrated care programmes, which would provide insight into why and where they are effective, is lacking [22]. Moreover, a recent review by Bautista et al., which has compared 200+ instruments of integrated care by looking at their measurement properties, found that most measurement properties of existing instruments need to be improved [23]. Hence the need for measurement, preferably based on sound evidence, of integrated care interventions which captures the complexity of integrated care as reflected in its multiple components and dynamic nature. This measurement will enable identification of potential problems in progress. The insight obtained into the relevant success factors will support the further development of integrated care.

A number of measurement models may be helpful in this activity. One model which has taken the complex dynamic and multiformity nature of integrated care into account and provides insight in the development process of integrated care, by describing four developmental phases, has been designed by Minkman et al. [12]. This model intends to be used as a quality management model for integrated care supporting the further development of integrated care practices. The model is, however, developed and used in the context of Dutch disease management programs and it is not clear how the approach extends to complex co-morbidities and long-term conditions. Furthermore, to transfer knowledge about successful integrated care interventions to other settings and thus support the development and scaling up of these interventions, it is important to consider the specific local conditions that influence the implementation and sustainability of a particular integrated care intervention [6].

A second model, has been developed by the B3 Action Group on Integrated Care of the European Innovation Partnership on Active and Healthy Aging (EIP on AHA) [24]. Unique to the B3-Maturity Model (B3-MM), is that it is derived from a pragmatic bottom-up approach with decision-makers involved in integrated care delivery from 12 European countries. These experts were interviewed about how healthcare systems are attempting to deliver more integrated care services to citizens. The rich collection of lessons learned are structured into 12 dimensions and reflect the various activities that need to be managed in order to deliver integrated care [25]. The B3-MM explicitly focuses on the need to understand the context and environment (i.e. the regional delivery system and political and organisational environment) of integrated care interventions. The goal of the B3-MM is to provide a self-assessment tool for European regions to assess their maturity in the provision of integrated care, thereby revealing strengths and areas for improvement. To demonstrate B3-MM’s full potential as a tool for measuring the maturity of integrated care, testing and validation of the tool is, however, needed.

Validity is an important feature in selecting or applying an instrument and is defined as ‘the degree to which an instrument truly measures the construct(s) it purports to measure’ [26]. The construct is a well-defined and precisely demarcated subject of measurement. Three forms of validity can be determined: content validity, criterion validity and construct validity [27]. The purpose of a content validation study is to determine whether the instrument adequately represents the construct under study [28]. Assessing content validity of an instrument is useful as it provides information on the representativeness and clarity of each item of the instrument. Furthermore, substantial suggestions are obtained to improve the measure, saving numerous revisions of the untested measure through several pilot evaluation studies [29]. The improved instrument can then be used in a pilot study to assess other psychometric properties.

This study has two objectives. As part of the European project SCIROCCO [30], it is to test the appropriateness of B3-MM’s dimensions, maturity indicators and assessment scale. Moreover, it also aims to test the content validity of B3-MM. In doing so, this paper reports on the following research questions fundamental to the study:

1. How is maturity of integrated care measured by instruments identified in the scientific and non-scientific literature?
2. How relevant are the dimensions, maturity indicators and assessment scale of B3-MM according to international experts in the field of integrated care?

## Theory and methods

### Theoretical background

#### Strategy for expansion of integrated care

Since 2013, the B3 Action Group on Integrated Care of the EIP on AHA has been collecting good practices in integrated care in Europe [25]. The extensive collection of good practices has provided a better understanding of the existing solutions, resources and expertise in integrated care delivery. However, the collected good practices are often limited to a particular pilot, project or region and the ambitions of the EIP on AHA and the B3 Action Group in particular is to promote the scaling up of these local initiatives throughout Europe [25]. In order to meet this ambition, the challenge remains on how to best leverage the existing evidence and support scaling up of good practices in Europe.

There is an increase in literature describing frameworks for scaling health interventions, the majority of which has an explicit focus on scaling up health actions in low- and middle-income country contexts [31]. However, literature is sparse on scaling up long-term care innovations in developed healthcare systems [32]. A 2016 study by Nolte et al. [4], examining three pilots in integrated care delivery, focussing on their development, implementation and sustainability including how they impacted the wider system context. It showed that the wider dissemination of the projects studied occurred in an incremental and somewhat random way. To guide the necessary transformation a formal strategy for expansion is needed [4], preferably based on sound evidence.

#### The B3 Maturity Model

**Figure 1:**
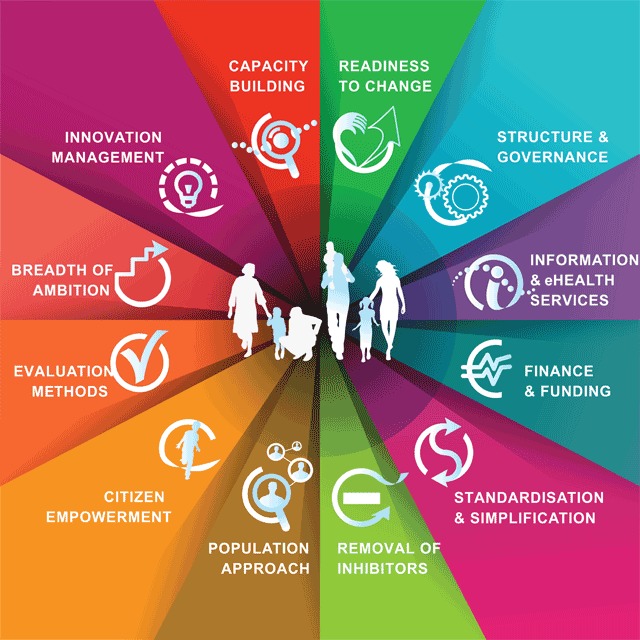
Dimensions of the B3-MM (retrieved from: http://www.scirocco-project.eu/maturitymodel/).

Recognizing the need for a structured approach which could stimulate path-breaking changes towards more sustainable health and care systems, partners of the B3 Action Group on Integrated Care of the EIP on AHA developed the B3-MM (to obtain a more standardised approach for scaling-up integrated care throughout Europe). The B3-MM is derived from an observational study, based on interviews with decision-makers in 12 European countries, or regions within a country, responsible for health care (namely Attica, the Basque Country, Catalonia, Galicia, Northern Ireland Saxony, Medical Delta, Olomouc region, Puglia region, Scotland, Skane, and South Denmark) over 18 months in 2014–2015. The interviews involved asking three sets of questions, to uncover i) the extent of integration already achieved, ii) the journey taken to get to this point, and iii) a view of future plans and investments. The outcomes of the study served as the baseline for the development of the B3-MM [25]. The maturity model intends to serve as a self-assessment tool for regions or health care systems that aim to assess progress along 12 dimensions. These dimensions reflect the various aspects to be managed in order to deliver integrated care (Figure 1) [30]. By considering each dimension, assessing the current situation within a healthcare system, and allocating a measure of ‘maturity’ (on a 0–5 scale), it should become possible to develop a simple graphical representation (i.e. spider diagram) of maturity level of the region/healthcare system, including its strengths and weaknesses in the path towards integrated care delivery. Using these insights, and comparing the findings with other regions that have conducted the same self-assessment process, should inform the complementary regions or healthcare systems about the possibility to progress with further knowledge transfer activities in order to improve their maturity in integrated care. The process of information sharing on lessons learned could help other regions is expected to speed up the adoption of integrated care.

#### Maturity Models

The art of measuring maturity displayed in the Capability Maturity Model (CMM) was introduced in the mid 80’s by the Software Engineering Institute (SEI) – Carnegie Mellon University [33]. Since then, several disciplines, especially those in the field of information systems, have successfully used maturity models as a way to assess the value and improve the competence of organisations [34]. A maturity model is regarded as a conceptual model which is characterised by the display of several maturity levels representing the developmental capabilities of organisations [35]. A limited number of studies have adapted these maturity models to the healthcare domain or proposed healthcare specific maturity models [36]. Blondiau et al. [37, p. 2] state that these healthcare specific maturity models show “a staged representation of an actual state in relation to a potentially achievable goal state and a description of steps required to achieve this objective.” Several models already exist, including the HIMSS Analytics Continuity of Care Model [38], TEMPEST (an Integrative model for health technology assessment) [39], and Maturity Matrix to Support Health and Social Care Integrated Care Partnerships [40]. However, the three above mentioned models tend to be either too simplistic [38,40], focusing for example only on the functionality of IT systems, or rather complex, given that they offer a volume of different factors to consider [39].

By regarding the development, implementation and scaling up of health innovations as a multi-stage process [41], the rationale of the B3-MM being a maturity model should be found in the evolution of integrated care services. The B3-MM displays the development of a regional system on several dimensions to achieve integrated care delivery. The B3-MM is considered to be a practical model, and thus easy to use, to comprehensively assess the maturity of the progress of a regional system thereby uncovering gaps and areas for improvement in the development of integrated care delivery. The insights gained by employing the B3-MM are intended to be used as a starting point from which regions with complementary strengths and weakness could be matched and start to share their lessons learned on specific areas. In doing so, the B3-MM is intended to be used to guide the process on how developments in one jurisdiction can inform developments in other regions. This process allows a tailored approach for regions in their journeys towards (more) integrated care as the regions can decide for themselves which areas attention should be paid to in order to speed up the adoption of or the development towards integrated care. Depending on the local needs of the regions, these tailored approaches can operate at the several dimensions and levels of integrated care [8]. Sensitivity to the differences between countries in terms of local needs and context to obtain a tailored approach for achieving progress in integrated care is important, as external contextual factors have been found to support the successful implementation of an integrated care model [42].

### Methods

In this study, we conducted a literature review and a Delphi study to test the content validity of B3-MM as instrument to measure the level of maturity of integrated care. Content validity can be determined using both quantitative or qualitative methods [43]. A qualitative approach consists of an accurate analysis of the representativeness and clarity of items in the literature and by consultation of experts [44]. Evidence of content validity is usually obtained by having knowledgeable people look at the test items and make judgments about the appropriateness of each item and overall coverage of the domain [45].

#### Literature review

A review was conducted to identify articles, papers and/or reports focusing on measures and instruments of the maturity of integrated care. Moreover, we were interested in describing and comparing the dimensions, indicators, measurement scales, and the psychometric property content validity of the selected measures and instruments.

The literature search consisted of two parts. For the first part, we built on the work of Bautista et al. [23] who recently conducted a systematic review in MEDLINE/PubMed on measurement properties of instruments measuring integrated care. The authors selected articles from the systematic literature review which focused on measures and instruments of the development of integrated care with indications of “maturity”, “phase”, “level”, or “degree” of integrated care.

To broaden the search for articles, a narrative review was undertaken. In narrative reviews, the authors have the objective to identify, evaluate and synthesize what is already known about a topic [46]. The preliminary search started in the electronic databases Cochrane, Google, Google Scholar, GreyLit, IDEA and OpenGrey using a combination of search terms, as shown in Table 1. The final search was restricted to the databases which retrieved adequate hits; Google (Filter: English only), Google Scholar and IDEA. The search terms used included terms referring to the construct, integrated care, and terms referring to an instrument. We used the terms from the study of Bautista et al. [23] who derived the terms from the work of Uijen et al. [47] and Terwee et al. [48]. We added search terms indicating a measurement feature of an instrument. The final key terms used in the ultimate search strategy are presented in Table 5.

**Table 1:** Search terms used in narrative literature review.

Component	Terms	Remarks

Construct	Integrated care, coordination of care, continuity of care, patient centered care	Based on the work of Uijen et al. [47] modified by Bautista et al. [23]
Instrument	Questionnaire, measure, survey, instrument	User-defined based on Terwee et al. [48]
Feature	Degree, maturity model, level, phase	Terms reflecting “maturity”

To be included in the review, we used the two eligible article criteria:

1. availability of full-text English document; (Due to the large number of hits, we limited the search to that of English language only when possible);
2. description of items/constructs/measurement scales of measures and/or instruments on the maturity of integrated care.

First, one researcher (LG) screened the titles and abstract of the articles from the main search in the three databases to identify articles for full text read. Two researchers (LG and HV) independently screened the full texts to select articles to be included in the final review.

#### Data extraction and analysis of the literature review

Data were extracted by looking for descriptions on dimensions, indicators, and measurement scales in the selected articles which matched with the 12 dimensions, maturity indicators and assessment scale of the B3-MM. We marked all matching items and listed them in a table developed in MS EXCEL. Descriptions on dimensions, indicators or measurement scales in the selected articles which did not match, but which could nevertheless provide an addition to B3-MM, were also identified. Furthermore, we evaluated the overall quality of the measurement property content validity (definition in **Box 1**) of the instruments identified in the narrative review based on the criteria used by Bautista et al. [23].

**Box 1: Definition of measurement property content validity (adapted from Uijen et al.) [47]**

Content validity: the degree to which the content of an instrument is an adequate reflection of the construct to be measured.

In quality assessment, there is an important distinction between the quality of a study on measurement properties and the quality of an instrument [49]. In the article by Bautista et al. [23], the quality assessment of the studies and the instruments is guided by the COnsensus-based Standards for the selection of health status Measurement INstruments (COSMIN) [26,28,50,51]. In this study, the overall quality of the content validity for the instruments was assessed by the researchers (LG and HV) using the criteria for the levels of evidence and overall assessment of measurement properties of instrument (Table 2) by determining four factors [23,27,47,52]. The first factor includes the number of validation studies per instrument. Snowball sampling and hand searching in Google and Google Scholar were performed to identify validation studies on the retrieved instruments from the narrative search. The second factor concerns the assessment of methodological quality of the studies relating to content validity. This assessment was based on the criteria of the COSMIN checklist [28] using the four-point scale in the COSMIN checklist. A study was rated as poor, fair, good, or excellent according to its measurement property content validity. The third factor is about the assessment of the direction of results of the measurement property content validity (whether positive or negative). This was rated using the modified criteria as presented in Table 3 [47]. The fourth factor entails the assessment of the consistency of several studies on the same instrument.

**Table 2:** Criteria for the level of evidence and overall assessment of measurement properties.

Criteria^a^	Overall assessment	Level of evidence

Consistent findings in multiple studies of good methodological quality OR in one study of excellent methodological quality	+++ or – – –	Strong
Consistent findings in multiple studies of fair methodological quality OR in one study of good methodological quality	++ or – –	Moderate
One study of fair methodological quality	+ or –	Limited
Conflicting findings from multiple studies	+/–	Conflicting
Only studies of poor methodological quality OR only indeterminate results from multiple studies regardless of methodological quality	?	Unknown
Measurement property not assessed	0	Not assessed

^a^ Adapted from Uijen et al. [47].

**Table 3:** Criteria for rating the adequacy of the reported measurement properties.

Measurement property	Reported Result	Quality criteria [47]

Content validity	+	The target population considers all items in the questionnaire to be relevant AND considers the questionnaire to be complete
	?	No target population involvement
	–	The target population considers items in the questionnaire to be irrelevant OR considers the questionnaire to be incomplete
	0	Did not assess content validity

#### Delphi study

To test the appropriateness of the items of the B3-MM to measure maturity of integrated care, an international Delphi study was performed. The Delphi technique is a widely used research method in healthcare research, which consists of “a series of data collection ‘rounds’ to capture and structure the knowledge and opinions of a ‘panel’ of participants on a topic with which they are perceived to have expertise” [42, p. 208].

##### Selection of experts

The experts were selected on basis of relevant experience in scientific research or having a practical background (medicine, nursing, managerial, policy making) with relevant experience in the development, implementation and/or monitoring of integrated care interventions. An overview of the type of experts who were invited to the first round of the Delphi survey is presented in Table 4. A total number of 55 experts received the email invitation that included information about the purpose and process of the study and a link to an online version of the questionnaire in SurveyMonkey. We asked the experts to commit their participation in two planned Delphi rounds.

**Table 4:** List of experts in the first Delphi round.

Types of experts	Number of experts selected	Experts retrieved from

Corresponding/first author of scientific articles (researchers with experience in the measurement or development of integrated care)	10	Articles included in the literature review used in the study
Experts with practical experience in the development, implementation and/or monitoring of integrated care interventions	10	SCIROCCO consortium partners*
Experts from the B3 Action Group on Integrated care	11	SCIROCCO consortium partners*
Experts with experience in the field of Information and eHealth services in the field of integrated care	10	SCIROCCO consortium partners*
Members of the SCIROCCO advisory board	5	SCIROCCO consortium partners*
Researchers with expertise in measurement of development of integrated care	9	A convenience sample provided by one of the researchers

* Basque Country (ESP), Norrbotten Lans Landsting (SE), Puglia region (IT), Olomouc region (CZ) and Scotland (UK).

#### First Delphi round

In the first Delphi round, experts were asked to rank the relevance of the dimensions, indicators and assessment scale of B3-MM to assess maturity of integrate care on a 9-point Likert scale (1 = Extremely irrelevant to 9 = Extremely relevant). The Likert scale corresponds to the conventional format used for comparative assessment and prioritisation of different health options (such as technologies) [54]. The survey started with general questions (including age, country of employment, disciplinary field, and years of experience) and continued with statements on the relevance of components of the B3-MM. These statements were presented in three different parts. The first part (A) considered statements on the relevance of the 12 dimensions (12 statements); the second part (B) reflected statements on the relevance of each indicator on the maturity scales on every dimension used in B3-MM (72 statements); the third part (C) included statements on the relevance of the assessment scale (12 statements). The survey concluded with a set of open-ended questions. One question included a possible addition to the assessment scale which was retrieved from the literature review on existing tools and measures by Ahgren & Axelsson [55]. Experts were asked to assess if a part of the measurement scale used in the tool of Ahgren & Axelsson [55], referring to the assessment of both the actual rank and the optimum rank of integration, could provide a meaningful addition to the assessment scale as used in B3-MM. Finally, experts were asked if they had any additional comments/suggestions on B3-MM or the survey. The survey was anonymised and a single reminder email message was sent to the experts. To diminish potential misunderstandings concerning the interpretation of the survey, the first survey round was pre-tested by two researchers (YM, LB). The survey was adjusted to reflect their feedback, including a clearer introduction to part B and C of the survey about statements on the assessment of the relevance of each indicator and scale. Experts were invited to the first survey in three different streams due to the arrival of late responses to the call for experts. The respondents were given one and a half weeks to complete the first survey.

#### Second Delphi round

The items for which insufficient agreement was found were rephrased by partners of the SCIROCCO consortium and presented to experts in the second Delphi round. A total number of 44 experts were invited to the second round. They were asked to reassess the relevance of the refined maturity indicators of the B3-MM items on the same 9-point Likert scale. Furthermore, they were asked to what extent they considered the addition to the assessment scale relevant by assessing both the actual rank and the optimum rank of integration using the B3-MM. Again, the experts were asked if they had any comments on the rephrased items or feedback on the survey. The second invitation included a report on the outcomes of round one of the Delphi exercise, including (1) a median agreement rating (interquartile range (IQR)) on every statement, (2) the level of agreement among the experts, (3) the level of disagreement among experts, and (4) whether consensus had been achieved. After discussion among the researchers and members of the SCIROCCO consortium, it was decided to exclude certain participants from the exercise due to a perceived conflict of interest: five members from the SCIROCCO advisory board (who had not participated in the first round of the exercise) and two active members of the SCIROCCO consortium (who had participated in the first round) were excluded from further participation. Again, the experts were given one and a half weeks to complete the second Delphi round.

#### Third Delphi round

The third Delphi round was conducted to explore the level of agreement among experts on the items with insufficient agreement in the second Delphi round. These items were rephrased by partners in the SCIROCCO consortium. Using the same 9-point Likert scale, experts were asked to reassess the relevance of the refined features of the B3-MM. The 13 experts who participated in the second round were re-invited to participate in the third Delphi round. The invitation included a report on the outcomes of the previous round, including (1) a median agreement rating (IQR) on every statement which was included in the second round, (2) the level of agreement among the experts, (3) the level of disagreement among the experts, and (4) whether consensus had been achieved. Experts were given the opportunity to provide feedback on the survey. Due to the project’s deadlines and the small number of statements in the third round, experts were given one week to complete the last round.

#### Data analysis of the Delphi study

Before conducting the Delphi survey, we defined the conditions of agreement among experts to be applied during the three Delphi rounds. In order to determine consensus within a Delphi study, many studies use a predefined level of agreement among the experts [56]. However, no standard threshold for consensus is offered by the literature [53], with thresholds for consensus ranging from 55%–100% [57]. In our study we decided on using a 75% cut off point, which is suggested and used by several studies to clearly differentiate the consensus and non-consensus results [53,58,59].

**Figure 2:**
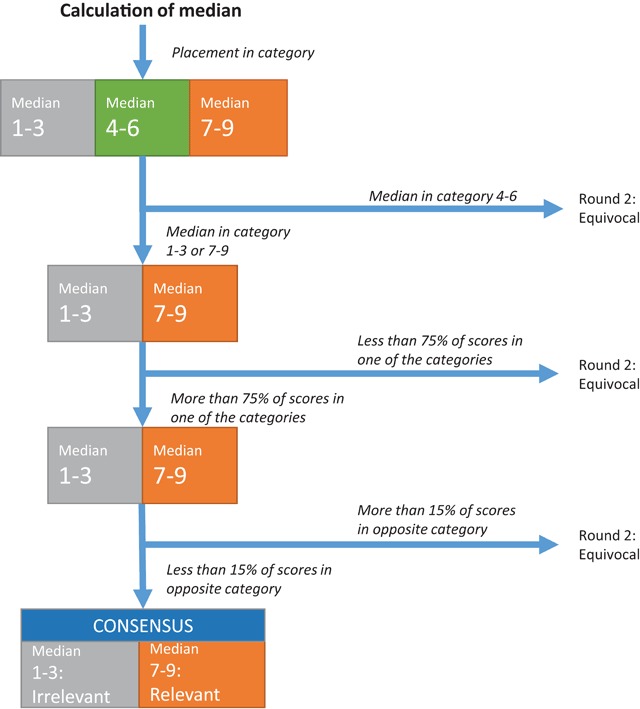
Flowchart calculation of consensus.

The 9-point scale was classified in three options; 1–3 as irrelevant, 4–6 as equivocal and 7–9 as relevant. The experts’ overall consensus on every statement on the items in the B3-MM was analysed using the median of the group’s scores and the “level of agreement” reached. Agreement among the experts on every statement on the items in the maturity model was reached when more than 75% of the experts’ ratings were within the same three-point range (that is, 1–3, 4–6, or 7–9) as well as the observed median. Several studies use a cut-off point of more than 75% of participants scoring 7 to 9, and include the condition (without disagreement) that less than 15% of the participants should have a scoring of between 1 to 3 [60,61]. In this study, we used the 75% threshold for reaching consensus, including the condition that less than 15% of the participants should have a scoring in the opposite range of that scale (Figure 2). Furthermore, the qualitative comments derived from the answers to the question on the optimum and actual rank, and the comments/suggestions on B3-MM and feedback on the survey were analysed using a qualitative approach. Analysis were performed in MS Excel. Under Belgium law no ethical approval is required to interview experts as part of a Delphi panel.

## Results

### Literature review

**Figure 3:**
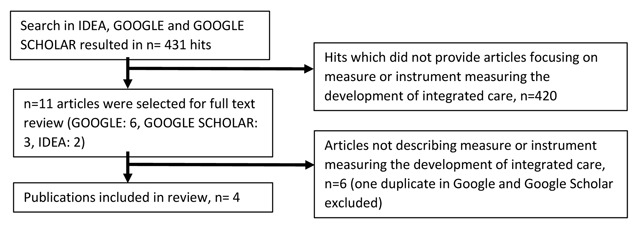
Flowchart narrative review process.

Out of the 300 articles included in the study of Bautista et al. [23] a total of seven articles were selected for our review [55,62,63,64,65,66,67]. From the narrative search, an additional number of four articles were retrieved. One duplicate full-text article from Bainbridge et al. [68] selected from Google and Google Scholar described a framework to guide evaluation and a more recent study was available describing the instrument which was based on this framework [69]. We included this article in the review instead of the initial full-text article retrieved. Details on the review process are presented in Figure 3. The combination of final search terms used for each database, date searched and the hits retrieved are shown in Table 5. The characteristics of the selected articles are shown in Appendix A.

**Table 5:** Oversight narrative review search terms and hits.

Database	Final used search term combination/string	Date search	Hits	Filter	Selected articles based on title/abstract	Selected articles after full text selection	Grey literature	Peer-reviewed literature	Total included in review

IDEA	“integrated care”	26-7-2016	126	None	2	1	0	1	1
GOOGLE	integrated care or coordination of care or continuity of care or patient centered care and measure or instrument or survey or questionnaire and degree or maturity model or level or phase	1-8-2016	164	English only	6 (1 duplicate with Google Scholar)	2	0	2 (1 dissertation)	2
Google Scholar	(“integrated care” or “coordination of care” or “continuity of care” or “patient centered care”) and (measure or instrument)	29-7-2016	141	None	3 (1 duplicate with Google)	1 (Retrieved from the article of Bainbridge et al. [68])	0	1	1

Overall, there is considerable similarity between the content of the original B3-MM model, and the instruments described in the articles selected from the literature review. All 12 dimensions and the related indicators described by the B3-MM corresponded with the content of the 11 retrieved articles (Table 6). Two dimensions of the B3-MM (“Information and eHealth services” and “Breadth of Ambition”) were described by all 11 articles. The content of over half of the articles matched with descriptions of ten of the dimensions. Less than half of the selected articles described items which matched with the two dimensions, “Population Approach” and “Innovation Management”. Apart from looking for matching descriptions, we searched for the use of possible dimensions, indicators or measurement scales which are not part of B3-MM (as it existed at the start of the project), and could complement or refine the B3-MM. One measurement scale was found which could provide a complement to the B3-MM: it was retrieved from the study of Ahgren & Axelsson [55]. They use a measurement model that can be used to evaluate the degree of integration, focusing on the functional aspects of clinical integration in arrangements of integrated care. In their model, the actual and the optimum rank of integration between units of the health authority are rated. This measurement feature could provide an extension to the B3-MM. It would enable the B3-MM to assess both the actual rank and the optimum rank of integration. Thus, it would provide a contextual explanation for the current situation in integrated care delivery while measuring the maturity of integrated care. This issue was further explored in the first two rounds of the Delphi study.

**Table 6:** Overview of articles matching descriptions with B3-MM.

Dimensions and related indicators as described in B3-MM [30]	Number of article(s) [Reference]

1. Readiness to change to enable more integrated care	8 [12,55,64,65,67,69,70,71]
1.1 No acknowledgement of crisis	
1.2 Crisis recognized, but no clear vision or strategic plan	
1.3 Dialogue and consensus-building underway; plan being developed	
1.4 Vision or plan embedded in policy; leaders and champions emerging	
1.5 Leadership, vision and plan clear to the general public; pressure for change	
1.6 Political consensus; public support; visible stakeholder engagement	

2. Structure and Governance	6 [12,55,64,67,69,71]
1.1 No overall attempt to manage the move to integrated care	
1.2 Change underway, but with fragmented organisations & plans	
1.3 Formation of task forces, alliances and other informal ways of collaborating	
1.4 Governance established at a regional or national level	
1.5 Roadmap for a change programme defined and broadly accepted	
1.6 Full, integrated programme established, with funding and a clear mandate	

3. Information and e-Health Services	11 [12,55,62,63,64,65,66,67,69,70,71]
1.1 No connected health services, just isolated medical record systems	
1.2 No integrated services used, only pilots/local services	
1.3 eHealth deployed in some areas, but limited to specific organisations or patients	
1.4 Voluntary use of regional/national eHealth services across the healthcare system	
1.5 Mandated or funded use of regional/national eHealth infrastructure across the healthcare system	
1.6 Universal, at-scale regional/national eHealth services used by all integrated care stakeholders	

4. Standardisation & Simplification	7 [12,64,65,67,69,70,71]
1.1 No systematic attempt to standardise the use of citizen health & care data, or to simplify systems in use	
1.2 Debate on information standards (e.g., coding, formatting); exploration of options for consolidating ICT	
1.3 A recommended set of agreed information standards at local level; a few local attempts at ICT consolidation	
1.4 A recommended set of agreed information standards at regional/national level; some shared procurements of new systems at regional/national level; some large-scale consolidations of ICT underway	
1.5 A unified set of agreed standards to be used for system implementations specified in procurement documents; many shared procurements of new systems; consolidated data centres and shared services widely deployed	
1.6 A unified and mandated set of agreed standards to be used for system implementations fully incorporated into procurement processes; clear strategy for regional/national procurement of new systems; consolidated datacentres and shared services (including the cloud) is normal practice.	

5. Finance & Funding	8 [12,55,63,64,67,69,70,71]
1.1 No special funding allocated or available	
1.2 Fragmented innovation funding, mostly for pilots	
1.3 Consolidated innovation funding available through competitions/grants for individual care providers	
1.4 Regional/national (or European) funding or PPP for testing and for scaling-up	
1.5 Regional/national funding for scaling-up and on-going operations	
1.6 Secure multi-year budget, accessible to all stakeholders, to enable further service development	

6. Removal of inhibitor	7 [12,55,64,67,69,70,71]
1.1 All projects delayed or cancelled due to inhibitors	
1.2 Some projects delayed or cancelled due to inhibitors	
1.3 Process for identifying inhibitors in place	
1.4 Strategy for removing inhibitors agreed at a high level	
1.5 Solutions for removal of inhibitors developed and commonly used	
1.6 High completion rate of projects & programmes; inhibitors no longer an issue for service development	

7. Population Approach	5 [12,66,69,70,71]
1.1 No consideration of population health in service provision	
1.2 A population focus of risk stratification but no risk stratification tools	
1.3 Individual risk stratification for the most frequent service users	
1.4 Group risk stratification for those who are at risk of becoming frequent service users	
1.5 Population-wide risk stratification started but not fully acted on	
1.6 Whole population stratification deployed and fully implemented.	

8. Citizen empowerment	7 [12,62,65,66,67,69,71]
1.1 No systematic plan for empowerment	
1.2 Citizens are not involved in decision-making processes and do not participate in the co-design of their services	
1.3 Policies to support citizens’ empowerment and protect their rights, but may not reflect their real needs	
1.4 Incentives and tools to motivate and support citizens to co-create health and participate in decision-making processes	
1.5 Citizens are supported and involved in decision-making processes, and have access to information and health data	
1.6 Citizens are involved in decision-making processes, and their needs are frequently monitored and reflected in service delivery and policy-making.	

9. Evaluation methods	6 [12,64,67,69,70,71]
1.1 No routine evaluation	
1.2 Evaluation exists, but not as a part of a systematic approach	
1.3 Evaluation established as part of a systematic approach	
1.4 Some initiatives and services are evaluated as part of a systematic approach	
1.5 Most initiatives are subject to a systematic approach to evaluation; published results	
1.6 A systematic approach to evaluation, responsiveness to the evaluation outcomes, and evaluation of the desired impact on service redesign (i.e. a closed loop process)	

10. Breadth of ambition	11 [12,55,62,63,64,65,66,67,69,70,71]
1.1 No level of integration	
1.2 Services in silos; the citizen or their family as the integrator of services	
1.3 Integration within the same level of care (e.g., primary care)	
1.4 Integration between care levels (e.g., between primary and secondary care)	
1.5 Integration includes both social care service and health care service needs	
1.6 Fully integrated health & social care services	

11. Innovation management	4 [12,64,69,71]
1.1 No plan for innovation management	
1.2 Isolated innovations across the region/country, but limited visibility	
1.3 Innovations are captured and published as good practice	
1.4 Innovation is governed and encouraged at a region/country level	
1.5 Formalised innovation management process in place	
1.6 Extensive open innovation combined with supporting procurement & the diffusion of good practice.	

12. Capacity building	8 [12,62,63,64,65,67,69,71]
1.1 No plan for capacity-building	
1.2 Single organisational initiatives engaged in process improvement	
1.3 Some mechanisms for sharing knowledge among organisations	
1.4 Systematic learning about IT; integrated care and change management	
1.5 Knowledge shared, skills retained and lower turnover of experienced staff	
1.6 A ‘learning healthcare system’ involving reflection and continuous improvement	

Regarding the assessment of the measurement property content validity of the instruments, we retrieved the data on the assessment of the overall quality rating score from the review of Bautista et al. [23] for the seven instruments selected from their study. Out of the 4 articles retrieved from the narrative review, three instruments were identified. No other validation studies on those three instruments were found by the hand searches and snowball sampling. In the dissertation included in the review concerning validation of the DMIC, three more validation studies were found. The results on the quality of the studies, the direction of results and the overall quality of the measurement property content validity of the instruments are shown in Table 7.

**Table 7:** Number of validation studies, the methodological quality of the studies, the direction (positive or negative) of results of the measurement properties and overall quality measurement property content validity score.

Instrument (data derived from Bautista et al. [23])	Author (name of first author only used) [reference]	Number of validation studies	Methodological quality of studies on content validity (COSMIN checklist [51])	Direction of results (Table 3) of measurement property content validity	Overall quality measurement property content validity score (Table 2)

Scale of Functional integration	Ahgren[55]	1	Fair	^a^	?
DELTA service user assessment	Ahgren [62]	1	Fair	^a^	+
Human Service Integration Measure	Browne [63]	1	Excellent	^a^	?
Unnamed1	Lukas [64]	1	Fair	^a^	+
Dual Diagnosis Capability in Health Care Settings (DDCHCS)	McGovern [65]	1	Not assessed	^a^	0
Patient Perceptions of Integrated Care Survey (PPICS)	Singer [66]	1	Fair	^a^	+
Unnamed2	Uyei [67]	1	Good	^a^	?
**Instruments (derived from the narrative review)**					

HCP integration survey	Bainbridge [69]	1	Fair	?	?
Unnamed3	Calciolari [70]	1	Fair	?	?
Development Model of Integrated Care (DMIC)		5			+++
	Minkman [12]		Excellent	+	
	Minkman [12]		Excellent	+	
	Minkman [12]		Excellent	+	
	Minkman [12]		Excellent	+	
	Longpré [71]		Fair	?	

^a^ Data on direction of results per instrument was summarised in the review of Bautista et al. [23]. No individual data per instrument was provided.

### Delphi study

#### First round

A total of 31 experts responded to the first survey round (response rate 56%). Three experts did not complete the survey. Furthermore, two experts were excluded due to a conflict of interest. The final analysis included 26 experts (84% completion rate). Reasons for non-participation included one delivery failure, one retirement, and two time constraints. The rest of the respondents did not provide reasons for not participating.

The outcomes on every statement of the first Delphi round are shown in Appendix B. Sufficient agreement was found among the experts on all 12 dimensions of B3-MM. Insufficient level of agreement was found for the first few indicators per dimension. Additionally, sufficient agreement was found on the assessment scale of the dimensions, except for the scale of “Innovation Management”.

Comments and suggestions with regard to the dimensions, indicators or assessment scale of B3-MM were provided by 17 out of 26 experts (65.4%). Although three experts provided positive comments with regard to the B3-MM, three other experts commented that some dimensions were unclear or that indicators in some of the dimensions were already covered by other dimensions. A total of five experts commented that some indicators/scales were ambiguous or contradictory and did not follow a logical structure. From the experts who provided feedback to the survey, two experts stated that the survey was difficult to understand and four experts did not fully understand the scale assessment in part C.

Regarding answers to the question about assessing the actual and optimum rank of integration, 22 out of 26 experts (84.6%) agreed that the actual and the optimum ranks of integration should be taken into account when measuring maturity of integrated care in a region or country.

#### Second round

A total of 14 experts responded to the second survey round (response rate 34%). One expert did not complete the survey. The final analysis included 13 experts (92.9% completion rate). One expert was not able to participate due to time constraints. The rest of the potential respondents did not provide reasons for not participating.

The outcomes for every statement of the second Delphi round are shown in Appendix C. Sufficient agreement was found among experts on the rephrased indicators, except for the two rephrased indicators, 8.2 and 9.1. Furthermore, 92.3% of the experts scored between 7–9 (median 7) in response to the question on the relevance of assessing both the actual rank and the optimum rank of integration, by applying B3-MM to provide a contextual explanation for the current situation while measuring maturity of integrated care. A total of six experts provided comments on the rephrased indicators. Three experts indicated that the rephrasing of the indicators was performed well. Furthermore, two experts emphasised that some of the rephrased indicators could still be made more explicit to distinguish these indicators clearly from the other indicators in their scale.

#### Third round

A total of 10 experts participated in the third Delphi round (response rate 76.9%). The rest of the potential respondents did not provide reasons for not participating. Sufficient agreement was found on both of the two rephrased indicators 8.2 and 9.1 (Appendix D).

The main characteristics of the expert group who participated in the first, second and Delphi round are presented in Table 8.

**Table 8:** Characteristics of experts in Delphi rounds 1, 2 and 3 (in % unless stated otherwise).

Characteristic	Category	Expert group first round (n = 26)	Expert group second round (n = 13)	Expert group third round (n = 10)

Age (year)	Min–Max	36–71	36–71	36–71
	Average (sd)	49.23 (11.73)	52.69 (13.22)	52.60 (13.43)
	<40	23.1	23.1	20
	40–50	30.8	23.1	30
	>50	46.2	53.8	50
Gender	Male	30.8	46.2	50.0
	Female	69.2	53.8	50.0
Country	Belgium	3.8	7.7	10
	Canada	7.7	7.7	10
	Czech Republic	3.8	7.7	10
	Finland	3.8	0	0
	Germany	3.8	0	0
	Italy	15.4	15.4	0
	Luxembourg	3.8	0	0
	Netherlands	7.7	0	0
	Netherlands and USA	3.8	7.7	10
	Portugal	7.7	7.7	10
	Spain	7.7	15.4	20
	Sweden	7.7	0	0
	UK	15.4	23.1	20
	USA	7.7	7.7	10
Professional Affiliation	Medicine	15.4	15.4	20
	Nursing	7.7	7.7	10
	Policy	7.7	15.4	0
	Managerial	15.4	23.1	20
	Research	46.2	30.8	40
	Other	7.7	7.7	10
Years of experience	<1	0	0	0
	1–5	38.5	23.1	30
	5–10	26.9	23.1	20
	>10	34.6	53.8	50

## Discussion

This study reports on the content validity of the B3-MM instrument, developed to measure the level of maturity of integrated care. The literature review and Delphi study allowed the assessment of the content validity of B3-MM and enabled the instrument to be enhanced. Following on from the review, the dimensions and indicators of the maturity model correspond to the items of instruments measuring maturity of integrated care in the academic literature. The results of the Delphi study showed that all the dimensions of the B3-MM are considered relevant by experts in the field of integrated care. Initially in the first Delphi round, there was insufficient agreement on the first few maturity indicators on every dimension whereas, after rephrasing the indicators during the second and third Delphi rounds, experts agreed that all the indicators were relevant for the assessment of the maturity of integrated care. As a result, B3-MM is considered to be a comprehensive instrument consisting of a wide range of dimensions applicable to the development of integrated care.

The items included in another instrument, called the DMIC, described in two articles, matched all the dimensions of the B3-MM [12,71]. While the DMIC is regarded as a validated generic quality management model for integrated care, the model was developed and widely used in the Netherlands [72]. In comparison, the B3-MM is of a wider scope, developed on basis of lessons learned in achieving integrated care by 12 different European regions.

In line with other studies [23,47], a variety in the constructs and elements measured by the selected instruments was observed in this study. Furthermore, the level of evidence on the overall quality of the measurement property content validity for only one out of ten instruments assessed in this study, was found to be strong. In their systematic review of measurement properties of care continuity instruments, Uijen et al. [47] indicated that these findings on the levels of evidence do not mean that the quality of the instruments is low, but rather that there is a need for high quality studies that can adequately assess the measurement properties and eventually the instrument quality. Moreover, out of the 300 articles retrieved in the literature review undertaken by Bautista et al. [23], only seven articles were included in this review. The need for high quality studies on measurement properties and the small number of selected articles indicates that the measurement of maturity in integrated care is not yet strongly developed in the academic literature. The complexity of the development, implementation and scale-up of the multi-stage process of integrated care makes the measurement of the maturity of integrated care a difficult exercise. However, if integrated care initiatives are to make a significant contribution to the transformation of health systems, solid measurement of the maturity of integrated care should become an essential element of their development. Measurement of the maturity of integrated care provides insight into both the problems experienced and the success factors that work when making progress on the development of integrated care services. It provides the knowledge needed to guide further development of integrated care initiatives in appropriate directions.

A few limitations need to be considered with regard to this study. The review was based on search terms derived from a systematic literature review which enabled a broad search in several databases. However, a first limitation of the narrative review is the focus on English language studies, which may have led to a language bias. A second limitation is that literature represent a large diversity of concepts (methods and measurements) concerning the measurement of integrated care [73]. Since “the definition and application of the concept of integrated care is influenced by the background and health care systems of the various authors” [12, p. 8], the data extraction from the literature conducted by the researchers is inevitably subjective. This is a disputable characteristic of any review that addresses complex interventions focusing on the items described for instruments in different contexts. A third limitation is that the review is susceptible to publication bias, although the search has been broadened to include literature found through various search engines. Concerning the overall assessment of the quality of the measurement property content validity we used data obtained from the review of Bautista et al. [23] on the score for the instruments and applied their criteria to the assessment of the instruments retrieved from our narrative review. The assessment is therefore subject to possible inconsistency although we tried to diminish this by discussing the assessment of the instruments among the researchers (LG and HV).

The Delphi technique has long been regarded as an appropriate research technique to reach consensus amongst groups of experts and has been widely applied in health and social studies [74]. However, there are currently no universally agreed criteria for the selection of experts; no directives on the minimum or maximum number of experts on a panel; and no firm guidelines on the correct number of rounds to be organised regarding the Delphi method; “rather the Delphi method appears to be related to common sense and practical possibilities” [46, p. 208]. Furthermore, the sample of the expert panels in the Delphi method are not being judged in terms of being representative samples for statistical purposes, but rather assessed on the qualities of the expert [75]. Although, we tried to reduce possible artefacts, a few limitations need to be considered for the Delphi study. To reach a reliable consensus in Delphi studies, it is important to establish a balance among the participants who represent a particular topic. The balance between the expert types who were recruited for the Delphi study and who participated in the first Delphi round was as follows: about half of the respondents who participated included researchers with experience in the measurement or development of integrated care. The other half consisted of a pool of experts who were recruited via the SCIROCCO consortium partners (i.e. a mix of experts with a practical experience in the development, implementation and/or monitoring of integrated care interventions, with experience in the field of Information and eHealth services or experts from the B3 Action Group on Integrated care).

The agreement found among the experts on the items of the B3-MM represents the majority opinion of the experts, yet, it does not mean the ‘right’ answers have been found [53]. The results may be biased due to the recruitment strategy that involved partners of the consortium; however, it may be expected that the experts provided their nuanced opinions garnered from their expertise. Furthermore, we provided room for the experts’ comments and suggestions as well as ensured that the Delphi rounds were completed anonymously without the influence of other panel members, to obtain a reliable and diverse collection of opinions. Additionally, a gradual decline in the number of experts participating in each Delphi round was observed. Although we provided experts with more than a week for responding and sent reminders, by asking for their participation in several rounds the Delphi technique asks much more dedication from respondents than does a simple survey, and the potential for low responses increases considerably [53]. A final limitation to the study is that a few expert respondents found the survey difficult to understand, which indicates that it is not evident that the instrument is easy to understand. The different backgrounds of the experts, concerning their fields of experience and origins (including variations in the types of health care systems, social values, and on-going health reform) may have also an influence on the way in which the instrument is interpreted. To obtain an adequate understanding of the instrument among its users, a clear manual explaining the meaning and application of the instrument would be desirable.

## Conclusion

Notwithstanding the pragmatic nature of the initial development of the B3-MM, this study is considered to be the first step to validate the B3-MM instrument to measure the maturity of integrated care. While today the B3-MM is a unique instrument based on existing knowledge and lessons learned in implementing integrated care, further research on its measurement properties is needed to enhance the quality of the B3-MM as instrument. The determination of the validity of an instrument measuring a construct is important. This further research on its measurement properties should preferably be guided by the COSMIN manual [51]. Moreover, in the SCIROCCO project, the use of the B3-MM instrument will be further explored as a tool to facilitate the exchange of good practices and scaling-up of integrated care processes in Europe. SCIROCCO will do so by testing a step-based strategy. In the first step, five participating health care regions in Europe are asked to use the tool for self-assessment of the of maturity level of the region or healthcare system in the path towards integrated care delivery. Based on the outcomes of the tool, the regions with complementary levels of maturity are matched. SCIROCCO will then organize twinning and coaching to facilitate shared learning among the regions. The SCIROCCO project will explore how matching the complementary strengths and weaknesses of regions can deliver two major benefits: a strong basis for successful twinning and coaching that facilitates shared learning and a practical support for the scaling up of good practices that promote active and healthy ageing and participation in the community [30]. As the B3-MM will be used as a starting point from which regions will be matched and shared learning will be facilitated, insight in the measurement properties of the tool is a prerequisite to ensure a valid and reliable assessment of the maturity level of the regional healthcare system. This will enable the more tailored process of achieving progress in the path towards integrated care for health care regions.

## Acknowledgements

We acknowledge the contribution of the following researchers participating in SCIROCCO:

- NHS 24 – Donna Henderson, Andrea Pavlickova
- UEDIN –Cristina-Adriana Alexandru, Stuart Anderson
- UVEG – Elisa Vaila Cotanda, Tamara Alhambra
- EHTEL – Marc Lange, Diane Whitehouse
- Kronikgune – Esteban de Manuel Keenoy, Jon Txarramendieta Suarez, Ane Fullaondo Zabala
- Osakidetza – Igor Zabala, Ainhoa Martin
- Ares Puglia – Francesca Avolio, Anna Elisabetta Graps
- FNOL – Zdenek Gutter, Michal Stybnar
- NLL – Lisa Lundgren, Ann-Charlotte Kassberg
- Vrije Universiteit Brussel – Yannick Marchal

## Additional Files

The additional files for this article can be found as follows:

10.5334/ijic.3063.s1 Appendix A Characteristics of articles identified.

10.5334/ijic.3063.s2 Appendix B Outcomes Delphi round 1.

10.5334/ijic.3063.s3 Appendix C Outcomes statements round 2.

10.5334/ijic.3063.s4 Appendix D Outcomes Delphi round 3.
