# Supplementary material for: An Instrument to Measure Maturity of Integrated Care: A First Validation Study
Source: Int J Integr Care. 2018 Jan 25;18(1):10. doi: 10.5334/ijic.3063 (PMC5853880; doi:10.5334/ijic.3063)
Supplement: Appendix A — Characteristics of articles identified. [file ijic-18-1-3063-s1.pdf]

Appendix A Characteristics of articles identified

	First author	Year	Country	Study design	Study objective	Instrument	Purpose of instrument	Type of measurement instrument	Items and measurement scale (items/scale)	Definition of maturity/development	Construct/items measured	Domain/ Construct classification	Type of respondent	Sample population	Context & setting	N, recruited	N, analysed	Health status
1	Ahgren [55]	2005	Sweden	Cross-sectional	To conceptualize and validate a model of measurement that can be used to evaluate the degree of integration in Local Health Care and similar arrangements of integrated care	Scale of Functional integration	To evaluate the degree of integration in local health care and similar arrangements of integrated care	Integration ranks were reported per health care unit based on consensus, data on integration were collected in self-assessment forms	One graphic scale (beginning with full segregation (rank=0) and ending in full integration (rank=100) is used to derive integration ranks for specific units identified (28 health care units)	''The model is based on a continuum of integration, extending from full segregation through intermediate forms of linkage, coordination and cooperation to full integration.''	Clinical integration	Other: Functional clinical integration	Health care providers	Integration ranks were reported per health care unit based on consensus	Primary care, secondary care, specialist care & community	25 (health care units)	18	(with disease) health care providers consider patient groups (with disease) of frequent occurrence
2	Ahgren [62]	2009	Sweden	Cross-sectional	To develop a model that can be used to assess the integration of welfare services from the perspective of the users and also to evaluate the results of these services.	DELTA service user assessment	To assess service integration	Questionnaire	Other: ordinal scales and open questions where used for the assessment. 32 items. The final questionnaire contained 16 structured questions linked to different ordinal scales.	''The process of integration requires adequate structural conditions, and together the process and the structure contribute to the outcome of integration. Together these dimensions can be used as a model to assess the integration of welfare services from the per-spective of the service users.''	Care integration (i.e. integration (dimensions of integration: process, structure and outcome)	Clinical integration	Patients (service users)	Services users of DELTA project (in Swedish delta means ''to participate''	Specialist care other local association for financial coordination between four different welfare institutions in vocational rehabilitation	552	386 (computed from total response rate)	(with disease) undergoing vocational rehabilitation
3	Browne [63]	2004	Canada	Cross-sectional	To propose a model and a measure of	Human Service integr	To quantify the extent, scope and	Telephone interview, web-	Not-item based, identifies specific	''This model can be used in any setting to identify the	Care integration (i.e. intra- and inter	Organisational integration	Health care providers	Agencies that participated	Other: Children programs:	27	Not applicable	Not applicable

					human service integration through strategic alliances with autonomous services as one way to achieve comprehensive health and social services for target populations	ation Measure	depth of integration as perceived by local service providers: a quantitative integration measure for each service and a total integration measure of the level of service integration -pilot test	form, in-person, or during workshops	services in the left hand column that are participating in programs of care. Rating scale, ordinal scale articulates a five domain continuum of increasing integration (0-4)	level of total and partial integration of human service along each of the three dimensions, or axes (services, goal and funding and other resources). It also permits analysis of the level of total and partial integration of human service across the three axes together, or across any two axes in any given setting. It identifies sectors or services missing from collaborative networks.''	sectorial service integration)			included groups from health, social, education, and community resources	Healthy babies, Healthy children HBHC. Early years program.			
4	Lukas [64]	2002	USA	Cross-sectional	To measure system integration in two ways by presenting empirically confirmed dimensions of system integration by providing a tool designed for ongoing use for managers.	Unnamed	To measure system integration	Survey	Rating scale. 11 initial domains. The central component of the scorecard is measuring system integration. Five scales represent aspects of system integration reported across all staff groups. Four additional integration system dimensions are based on	''The scorecard is intended to provide insight not only to the extent of system integration but also to the organizational features that lead to integration and to the system performance associated with system integration.''	Care integration	Combination (clinical, professional, system and normative)	Health care providers		Primary care, secondary care ,community, home-based care, nursing home	1042	1042	Healthy

									questions unique to the managers' version of the instrument.									
5	McGovern [65]	2012	USA	Cross-sectional	To assess the development and feasibility of DDCHCS to assess the level in which a care organization offers integrated behavioral health care services within the traditional medical settings	Dual Diagnosis Capability in Health Care Settings (DDCHCS)	To assess degree to which an organisation offers integrated behavioral health care service in both mental and substance abuse within traditional medical settings.	"field test" a new measure of organizational capacity of behavioral health service integration. All assessments included in the study were made by a pair of raters.	The instrument used in this study (DDCHCS, version 2.0) is composed of 36 benchmark items and organized by seven dimension. Rating scale.	''This measure, the Dual Diagnosis Capability in Health Care Setting (DDHCS) index, was designed specifically to assess the degree to which an organization offers integrated behavioral health care services, both mental health and substance use, within traditional medical settings. The DDCHCS is developed to be a practical benchmark measure of policy, practice and workforce dimensions which can serve to access integration at baseline, and then objectively guide quality improvement efforts.''	Care integration	Clinical integration	Others: organization level assessment	DDCHS assessment teams, assessment was conducted at the organization level	Primary care, community	13	13	Not applicable
6	Singer [66]	2013	USA	Cross-sectional	To develop and pilot a new instrument to measure integration of patient care from patients' perspective	Patient Perceptions of Integrated Care Survey	To measure level of integration from patient perspective	Survey	29 items. 7 dimensions. Dichotomous and Likert scale	''Our analysis of responses to a pilot survey developed to measure aspects of integrated care suggests a six-dimension measurement framework that is	Care integration	Combination (i.e. clinical and professional integration)	Patients	Patients with multiple chronic conditions	Primary care	527	527	(with disease) multiple chronic conditions

					s					largely consistent with our theoretical model and that can be used by health-system reformers to gauge the ongoing progress of their initiatives.''								
7	Uyei [67]	2014	South-Africa	Cross-sectional	To describe the development and results of a survey instrument that was designed to measure the degree to which TB and HIV services were jointly organized and delivered at clinics in Cape Town	Unnamed	To assess the delivery of TB, pre-art and art services in the clinic	Survey	35 items, rating scale. Scores ranged from 0 (strongly disagree) to 5 (strongly agree). A score of 5 corresponded to a stronger degree of service integration, whereas a score of 0 corresponded to a weaker degree of integration.	''Clinical integration refers to the extent to which diagnostic, treatment, care, rehabilitation and health promotion are concurrently or synchronically delivered to the patient. Services can be integrated in terms of structure (existence of formal guidelines and protocols regarding the practice of joint service delivery), process (behaviour and practice of delivering services) and culture (work place culture and personal identification with integrated service delivery). […] instrument designed to quantify the extent to which services were integrated in 33 clinics and presents the results of the	Care integration	Combination (i.e. functional, organisational and clinical integration)	Health care providers	Clinicians (doctors and nurses)	Public, other public clinics	77 (68.8% nurses, 31.2% doctors)	77	Not applicable

										survey.’’								
8	Bainbridge [69]	2014		Cross-sectional	Our objective was to assess horizontal integration within a PCN (palliative care network) from the perspectives of HCPs, guided by an empirically derived conceptual framework for the evaluation of integrated palliative care (Bainbridge et al., 2010).	HCP integration survey	To assess horizontal integration within a PCN from the perspectives of HCPs	Survey	60 items. Rating scale, Likert scale and dichotomous.	‘’Examination of interprofessional collaboration and functionality that suggest the extent to which horizontal integration exists among a care network of service agencies. Through this survey we were able to identify tenants of horizontal integration present in the study network of HCPs, but also gaps, many revealed specifically in response to the added items in the survey. The framework uses a Donabedian systems approach (Donabedian, 1966), specifically system structure, process and outcome. Our examination focused on the process level elements which constitute factors that are both indicative of and complementary to horizontal integration […].’’	Care integration	Professional integration	Health care providers	Nurses, palliative care physicians, personal support workers, therapists, social workers and other health professionals	Primary care, secondary care, specialist care & community	279	86	(with disease) health care providers providing palliative care
9	Calciolari [70]	2016	Italy	Cross-sectional	The present study had two main objectives. First, it tests the construct	Unnamed	To measure care integration	Questionnaire	Rating scale, Likert and binary scale, 24 items.	‘’A variety of international experiences support the interpretation of care integration as the result of a	Care integration	Combination (i.e. organisational, functional)	Directors	the Director of Social Services, Director of a Social	Hospital and community care services,	144 LHU	The dataset was constructed between July 1st,	The cover letter instructed the respondents to refer exclusively

					validity and reliability of a parsimonious instrument aimed to assess the phenomenon of care integration. Second, it proposed a conceptual framework designed to analyze the conditions or antecedents of integration, including the context and culture.					com-plex combination of contributing factors. We grouped the influential factors into four categories: contextual traits, transition management culture, organizational arrangements, and operating means. […] we found that integration depends on a range of factors at multiple levels, rather than being determined merely by the implementation of operating means.’’				Care District, Director of the Geriatrics Operative Unit, or the Director of the Department of Geriatrics			2011 and March 6th, 2012 and consists of 102 useful responses from 87 different LHUs, with a response rate of 60.4% and 19 Regions represented.	to ‘frail elderly patients’, who were defined as follows: (a) geriatric complex cases, suffering from poly-morbidity and presenting a high risk of adverse outcomes and (b) those who require comprehensive care, often being discharged from the hospital or a long-term care institution into a protected regime
10	Minkman (Chapter 8) [12]	2011	Netherlands	Validation study	Our aim in this study was to assess whether the four development phases were recognised by integrated care services in the Netherlands.	DMIC	To explore how local integrated care services are developed in the Netherlands, and to conceptualise and operationalise a development model of integrated	Literature study, Delphi methodology (expert judgements and comparison of the judgements in several rounds), concept mapping + a question	A three-part Excel-based questionnaire (A-C). Part A focussed on general information about the integrated care service. In part B the respondents rated the 89 elements of the DMIC in	‘’Four phases of development can be identified in integrated care practice. 1) initiative and design; 2) experimentation and execution; 3) expansion and monitoring; and 4) consolidation and transformation of the integration project.’’	Integrated care	Integrated care	Coordinators	Coordinators of integrated stroke, AMI and dementia services	Primary care, secondary care, specialist care and community	32 out of the 36 stroke services (89%), 9 out of the 12 AMI services (75%) and 43 out of the 50 dementia services (86%) participa	See previous box	(with disease) Coordinators of integrated stroke, AMI and dementia services

							care	naire	terms of relevance and existence in daily practice and where applicable since which year. In part C the descriptions of the four developme nt phases were presented and the respondent s each assessed their own developme nt phases.							ted.		
11	Longpré [71]	2015	Canada	Quantitative cross-sectional design	Our objectives are: 1) to determine the extent to which nursing interventions in care pathway implementation converge with demands for greater integration of care and services; and 2) to determine the extent to which nursing	Development model for integrated care (DMIC)	Survey	89 elements, yes/no	89 items. For each item, nurses were asked to answer yes–no questions relating to, on one hand, the relevance of the activity to their practice, and on the other, its presence, that is, to what extent they considered the activity to be prevalent (or valued)	‘’The activities associated with these dimensions are ranked by complexity, making it possible to identify, using an analysis grid, four phases of development in the integration process: 1) initiative and design; 2) experimentation and execution; 3) expansion and monitoring; and 4) consolidation and transformation of the integration project.‘’	Integrated care	Integrated care	Health care providers	All personnel with clinical functions except for patient care attendants (nursing assistant, technician, nurse clinician, counselor, navigator, liaison nurse, nurse practitioner) and management	Primary care, secondary care, specialist care and community	200	107	(with disease) nurses working in four different care pathways

					practice is at similar or different phases of development in the integration process in different				within their service. The second component, positioned 40 activities (out of the 89 integrative activities) considered the most significantly representative of the four phases of the process (10 activities per phase).					functions (coordinator, head nurse, assistant head nurse, director, manager).				
