# Supplementary material for: An Instrument to Measure Maturity of Integrated Care: A First Validation Study
Source: Int J Integr Care. 2018 Jan 25;18(1):10. doi: 10.5334/ijic.3063 (PMC5853880; doi:10.5334/ijic.3063)
Supplement: Appendix B — Outcomes Delphi round 1. [file ijic-18-1-3063-s2.pdf]

Appendix B Outcomes Delphi round 1

Statements	Round 1 (n=26)				
	Overall Experts Median and IQR	Agreement in 7-9 region (%)	Agreement in 4-6 region (%)	Agreement in 1-3 region (%)	Overall consensus
Dimensions					
1. Readiness to change	9 (1)	96.2	3.8	0	Relevant
2. Structure and Governance	8,5 (1)	100	0	0	Relevant
3. Information and e-Health Services	8 (2)	96.2	3.8	0	Relevant
4. Standardisation and simplification	8 (1)	92.3	3.8	3.8	Relevant
5. Finance and funding	8 (2)	100	0	0	Relevant
1. Removal of inhibitors	8 (1,25)	96.2	0	3.8	Relevant
2. Population approach	8 (4)	88.5	11.5	0	Relevant
3. Citizen empowerment	8 (4)	92.3	7.7	0	Relevant
9. Evaluation methods	8 (2)	88.5	11.5	0	Relevant
10. Breadth of ambition	8 (1)	92.3	7.7	0	Relevant
11. Innovation management	8 (1,25)	88.5	11.5	0	Relevant
12. Capacity building	8 (1)	88.5	11.5	0	Relevant
Indicators					
1. Readiness to change to enable more integrated care					
1.1 No acknowledgement of crisis	6.5 (5)	50	23.1	26.9	Equivocal (Round 2)
1.2 Crisis recognized, but no clear vision or strategic plan	7 (2.25)	61.5	23.1	15.4	Equivocal (Round 2)
1.3 Dialogue and consensus-building underway; plan being developed	8 (1)	80.8	19.2	0	Relevant
1.4 Vision or plan embedded in policy leaders and champions emerging	8 (2)	88.5	11.5	0	Relevant
1.5 Leadership, vision and plan clear to the general public; pressure for change	8.5 (1)	100	0	0	Relevant
1.6 Political consensus; public support; visible stakeholder engagement	8 (1)	88.5	11.5	0	Relevant
2. Structure and Governance					
2.1 No overall attempt to manage the move to integrated care	8 (4.25)	61.5	19.2	19.2	Equivocal (Round 2)
2.2 Change underway, but with fragmented organisations & plans	7.5 (2.25)	61.5	26.9	11.5	Equivocal (Round 2)
2.3 Formation of task forces, alliances and other informal ways of collaborating	8 (2)	88.5	11.5	0	Relevant
2.4 Governance established at a regional or national level	8 (2)	92.3	7.7	0	Relevant
2.5 Roadmap for a change programme defined and broadly accepted	8 (1.25)	92.3	7.7	0	Relevant
2.6 Full, integrated programme established, with funding and a clear mandate	8 (1)	84.6	11.5	3.8	Relevant
3. Information and e-Health Services					
3.1 No connected health services, just isolated medical record systems	7.5 (3.75)	61.5	15.4	23.1	Equivocal (Round 2)
3.2 No integrated services used, only pilots/local services	8 (2.5)	53.8	30.8	15.4	Equivocal (Round 2)
3.3 eHealth deployed in some areas, but limited to specific organisations or patients	8 (2)	73.1	15.4	11.5	Equivocal (Round 2)
3.4 Voluntary use of regional/national	7.5 (2)	69.2	23.1	7.7	Equivocal (Round 2)

eHealth services across the healthcare system					
3.5 Mandated or funded use of regional/national eHealth infrastructure across the healthcare system	8 (0.25)	100	0	0	Relevant
3.6 Universal, at-scale regional/national eHealth services used by all integrated care stakeholders	8 (2)	88.5	7.7	3.8	Relevant
4. Standardisation & Simplification					
4.1 No systematic attempt to standardise the use of citizen health care data, or to simplify systems use	8 (3.5)	61.5	15.4	23.1	Equivocal (Round 2)
4.2 Debate on information standards (e.g., coding, formatting); exploration of options for consolidating ICT	6.5 (3.5)	50.0	26.9	23.1	Equivocal (Round 2)
4.3 A recommended set of agreed information standards at local level; a few local attempts at ICT consolidation	7.5 (2)	73.1	19.2	7.7	Equivocal (Round 2)
4.4 A recommended set of agreed information standards at regional/national level; some shared procurements of new systems at regional/national level; some large-scale consolidations of ICT underway	8 (1.25)	76.9	15.4	7.7	Relevant
4.5 A unified set of agreed standards to be used for system implementations specified in procurement documents; any shared procurements of new systems; consolidated data centres and shared services widely deployed	8 (1.25)	84.6	11.5	3.8	Relevant
4.6 A unified and mandated set of agreed standards to be used for system implementations fully incorporated into procurement processes; clear strategy for regional/national procurement of new systems; consolidated datacentres and shared services including the cloud) is normal practice.	8 (2)	84.6	11.5	3.8	Relevant
5. Finance & Funding					
5.1 No special funding allocated or available	7 (2)	73.1	3.8	23.1	Equivocal (Round 2)
5.2 Fragmented innovation funding, mostly for pilots	8 (2)	73.1	15.4	11.5	Equivocal (Round 2)
5.3 Consolidated innovation funding available through competitions/grants for individual care providers	8 (1)	80.8	11.5	7.7	Relevant
5.4 Regional/national (or European) funding or PPP for testing and for scaling-up	8 (2)	84.6	15.4	0	Relevant

5.5 Regional/national funding for scaling-up and on-going operations	8 (1)	100	0	0	Relevant
5.6 Secure multi-year budget, accessible to all stakeholders, to enable further service development	8.5 (1)	84.6	11.5	3.8	Relevant
6. Removal of inhibitor					
6.1 All projects delayed or cancelled due to inhibitors	7.5 (4.25)	65.4	11.5	23.1	Equivocal (Round 2)
6.2 Some projects delayed or cancelled due to inhibitors	7.5 (4)	65.4	15.4	19.2	Equivocal (Round 2)
6.3 Process for identifying inhibitors in place	8 (2.25)	76.9	19.2	3.8	Relevant
6.4 Strategy for removing inhibitors agreed at a high level	8 (3)	73.1	23.1	3.8	Equivocal (Round 2)
6.5 Solutions for removal of inhibitors developed and commonly used	8 (1)	88.5	7.7	3.8	Relevant
6.6 High completion rate of projects & programmes; inhibitors no longer an issue for service development	8 (2)	80.8	15.4	3.8	Relevant
7. Population Approach					
7.1 No consideration of population health in service provision	7.5 (3)	57.7	23.1	19.2	Equivocal (Round 2)
7.2 A population focus of risk stratification but no risk stratification tools	7 (2)	53.8	38.5	7.7	Equivocal (Round 2)
7.3 Individual risk stratification for the most frequent service users	8 (2)	73.1	19.2	7.7	Equivocal (Round 2)
7.4 Group risk stratification for those who are at risk of becoming frequent service users	8 (1)	88.5	7.7	3.8	Relevant
7.5 Population-wide risk stratification started but not fully acted on	8 (1)	92.3	3.8	3.8	Relevant
7.6 Whole population stratification deployed and fully implemented.	8 (1)	84.6	7.7	7.7	Relevant
8. Citizen empowerment					
8.1 No systematic plan for empowerment	8 (4.5)	57.7	19.2	23.1	Equivocal (Round 2)
8.2 Citizens are not involved in decision-making processes and do not participate in the co-design of their services	8 (4.25)	61.5	15.4	23.1	Equivocal (Round 2)
8.3 Policies to support citizens' empowerment and protect their rights, but may not reflect their real needs	7 (3.25)	61.5	23.1	15.4	Equivocal (Round 2)
8.4 Incentives and tools to motivate and support citizens to co-create health and participate in decision-making processes	8 (1.25)	80.8	11.5	7.7	Relevant
8.5 Citizens are supported and involved in decision-making processes, and have access to information and health data	8 (1.25)	88.5	3.8	7.7	Relevant
8.6 Citizens are involved in decision-making processes, and their needs are frequently monitored and reflected in service delivery and policy-making.	8 (1.25)	84.6	7.7	7.7	Relevant
9. Evaluation methods					
9.1 No routine evaluation	8 (4.25)	19.2	15.4	65.4	Equivocal (Round 2)

9.2 Evaluation exists, but not as a part of a systematic approach	7.5 (2.75)	19.2	19.2	61.5	Equivocal (Round 2)
9.3 Evaluation established as part of a systematic approach	8 (2)	80.8	15.4	3.8	Relevant
9.4 Some initiatives and services are evaluated as part of a systematic approach	8 (2)	84.6	11.5	3.6	Relevant
9.5 Most initiatives are subject to a systematic approach to evaluation; published results	8 (1.25)	88.5	11.5	0	Relevant
9.6 A systematic approach to evaluation, responsiveness to the evaluation outcomes, and evaluation of the desired impact on service redesign (i.e., a closed loop process)	8 (1)	92.3	7.7	0	Relevant
10. Breadth of ambition					
10.1 No level of integration	7 (4)	61.5	19.2	19.2	Equivocal (Round 2)
10.2 Services in silos; the citizen or their family as the integrator of services	8 (3.5)	61.5	23.1	19.2	Equivocal (Round 2)
10.3 Integration within the same level of care (e.g., primary care)	8 (2.25)	76.9	19.2	3.8	Relevant
10.4 Integration between care levels (e.g., between primary and secondary care)	8 (2)	88.5	3.8	7.7	Relevant
10.5 Integration includes both social care service and health care service needs	8 (1)	88.5	3.8	7.7	Relevant
10.6 Fully integrated health & social care services	8 (1)	84.6	7.7	7.7	Relevant
11. Innovation management					
11.1 No plan for innovation management	7.5 (4)	57.1	19.2	23.1	Equivocal (Round 2)
11.2 Isolated innovations across the region/country, but limited visibility	7.5 (2.25)	53.8	26.9	19.2	Equivocal (Round 2)
11.3 Innovations are captured and published as good practice	8 (3)	73.1	19.2	7.7	Equivocal (Round 2)
11.4 Innovation is governed and encouraged at a region/country level	8 (1)	80.8	15.4	3.8	Relevant
11.5 Formalised innovation management process in place	8 (1)	80.8	11.5	7.7	Relevant
11.6 Extensive open innovation combined with supporting procurement & the diffusion of good practice.	8 (2)	84.6	11.5	3.8	Relevant
12. Capacity building					
12.1 No plan for capacity-building	7 (3.75)	65.4	11.5	23.1	Equivocal (Round 2)
12.2 Single organisational initiatives engaged in process improvement	7 (2.25)	61.5	23.1	15.4	Equivocal (Round 2)
12.3 Some mechanisms for sharing knowledge among organisations	8 (3)	73.1	15.4	11.5	Equivocal (Round 2)
12.4 Systematic learning about IT; integrated care and change management	8 (1.25)	80.8	11.5	7.7	Relevant
12.5 Knowledge shared, skills retained and lower turnover of experienced staff	8 (1)	88.5	3.8	7.7	Relevant
12.6 A 'learning healthcare system'	8 (1.25)	84.6	11.5	3.8	Relevant

involving reflection and continuous improvement					
Maturity scale (0-5)					
1. Readiness to change	8 (1.25)	84.6	11.5	3.8	Relevant
1. Structure and Governance	8 (2)	84.6	11.5	3.8	Relevant
2. Information and e-Health Services	8 (1.25)	84.6	11.5	3.8	Relevant
3. Standardisation and simplification	7 (1)	80.8	11.5	7.7	Relevant
5. Finance and funding	8 (2)	92.3	3.8	3.8	Relevant
6. Removal of inhibitors	8 (1)	88.5	7.7	3.8	Relevant
7. Population approach	8 (2)	84.6	7.7	7.7	Relevant
8. Citizen empowerment	8 (2)	80.8	11.5	7.7	Relevant
11 Evaluation methods	8 (2)	88.5	7.7	3.8	Relevant
10. Breadth of ambition	8 (2.25)	76.9	19.2	3.8	Relevant
11. Innovation management	7 (2)	73.1	23.1	3.8	Equivocal (Round 2)
12. Capacity building	8 (1.25)	80.8	15.4	3.8	Relevant
