# Supplementary material for: An Instrument to Measure Maturity of Integrated Care: A First Validation Study
Source: Int J Integr Care. 2018 Jan 25;18(1):10. doi: 10.5334/ijic.3063 (PMC5853880; doi:10.5334/ijic.3063)
Supplement: Appendix C — Outcomes statements round 2. [file ijic-18-1-3063-s3.pdf]

Appendix C Outcomes statements round 2

Statements	Round 2 (n=13)				
	Overall Experts Median and IQR	Agreement in 7-9 region (%)	Agreement in 4-6 region (%)	Agreement in 1-3 region (%)	Overall consensus
Rephrased indicators					
1. Readiness to change to enable more integrated care					
1.1 No acknowledgement of compelling need to change	8 (0.5)	100	0	0	Relevant
1.2 Compelling need is recognised, but no clear version or strategic plan	8 (1)	92.3	7.7	0	Relevant
2. Structure and Governance					
2.1 Fragmented structure and governance in place	8 (0)	100	0	0	Relevant
2.2 Recognition of the need for structural and governance change	8 (1)	84.6	15.4	0	Relevant
3. Information and e-Health Services					
3.1 Information systems are not designed to support integrated care	8 (2)	84.6	15.4	0	Relevant
3.2 Information & eHealth services to support integrated care are being piloted	8 (1)	92.3	7.7	0	Relevant
3.3 Information & eHealth services to support integrated care are deployed but there is not yet region wide coverage	8 (1)	100	0	0	Relevant
3.4 Information & eHealth services to support integrated care are available via a region-wide service but use of these services is not mandated.	8 (1)	92.3	7.7	0	Relevant
4. Standardisation & Simplification					
4.1 No standards in place or planned that support integrated care services	8 (2.5)	76.9	23.1	0	Relevant
4.2 Discussion of the necessity of ICT to support integrated care and of any standards associated with that ICT	8 (1)	84.6	15.4	0	Relevant
4.3 An ICT structure to support integrated care has been agreed together with a recommended set of information standards - there may still be local variations	8 (1)	92.3	7.7	0	Relevant
1. Finance & Funding					
5.1 No additional funding is available to support the move towards integrated care	8 (1)	84.6	7.7	0	Relevant
5.2 Funding is available but mainly for pilot projects and small scale implementation	8 (1)	100	0	0	Relevant
2. Removal of inhibitors					
6.1 No awareness of the effects of inhibitors on integrated care	8 (1)	84.6	15.4	0	Relevant
6.2 Awareness of inhibitors but no systematic approach to their management is in place	8 (1)	92.3	7.7	0	Relevant
6.4 Strategy for tackling inhibitors is agreed at a high level	8 (0)	100	0	0	Relevant
7. Population Approach					

7.1 Population health approach is not applied to the provision of integrated care services	8 (0.5)	92.3	7.7	0	Relevant
7.2 A population risk approach is applied to integrated care services but not yet systematically or to the full population	7 (1.0)	92.3	7.7	0	Relevant
7.3 Risk stratification is used systematically for certain parts of the population (e.g. high-use categories)	8 (0)	100	0	0	Relevant
1. Citizen empowerment					
8.1 Citizen empowerment is not considered as part of integrated care provision	8 (1.5)	100	0	0	Relevant
8.2 Citizens are consulted on integrated care services but are not involved in co-creation and coproduction of services	8 (2)	69.2	30.8	0	Equivocal
8.3 Citizen empowerment is recognized as important but effective policies to support citizen empowerment are still in development	7 (1)	84.6	15.4	0	Relevant
2. Evaluation methods					
9.1 Integrated care service evaluation is not seen as distinct from standard evaluation approaches	7 (2)	69.2	30.8	0	Equivocal
9.2 Recognition and development of evaluation designed to evaluate integrated care services	7 (1)	92.3	7.7	0	Relevant
10. Breadth of ambition					
10.1 Integrated services arise but not as a result of planning or the implementation of a strategy	8 (1.5)	76.9	23.1	0	Relevant
10.2 The citizen or their family may need to act as the integrator of services in an unpredictable way	8 (1.5)	84.6	15.4	0	Relevant
11. Innovation management					
11.1 No innovation management in place	8 (1.5)	76.9	23.1	0	Relevant
11.2 Innovation is encouraged but there is no overall plan	8 (1.5)	76.9	23.1	0	Relevant
11.3 Innovations are captured and there are some mechanisms in place to encourage knowledge transfer	8 (1.5)	84.6	15.4	0	Relevant
12. Initial indicators round 1: Capacity building					
12.1 Integrated care services are not included in capacity planning	8 (1.5)	76.9	23.1	0	Relevant
12.2 Some systematic approaches to capacity building for integrated care services are in place	8 (1)	84.6	15.4	0	Relevant
12.3 Cooperation on capacity building for integrated care is growing across the region.	8 (1)	100	0	0	Relevant
Statement on Actual and Optimum rank (Ahgren & Axelsson, 2005)	7 (1)	92.3	7.7	0	Relevant
