# Supplementary material for: An Instrument to Measure Maturity of Integrated Care: A First Validation Study
Source: Int J Integr Care. 2018 Jan 25;18(1):10. doi: 10.5334/ijic.3063 (PMC5853880; doi:10.5334/ijic.3063)
Supplement: Appendix D — Outcomes Delphi round 3. [file ijic-18-1-3063-s4.pdf]

Appendix D outcomes Delphi round 3

Statements	round 3 (n=10)				
	Overall Experts Median and IQR	Agreement in 7-9 region (%)	Agreement in 4-6 region (%)	Agreement in 1-3 region (%)	Overall consensus
8. Citizen empowerment					
Rephrased indicators second round					
8.2 Citizens are consulted on integrated care services but are not involved in co-creation and coproduction of services	8 (2)	69.2	30.8	0	Equivocal
Rephrased indicator third round					
8.2 Some citizen consultation on integrated care but not as part of a systematic approach to citizen empowerment for integrated care	8 (1)	90.0	10.0	0	Relevant
9. Evaluation methods					
Rephrased indicators second round					
9.1 Integrated care services evaluation is not seen as distinct from standard evaluation approaches	7 (2)	69.2	30.8	0	Equivocal
Rephrased indicator third round					
9.1 No evaluation of integrated care services is in place or in development	8 (0)	100	0	0	Relevant
